# The Important Interface Between Apolipoprotein E and Neuroinflammation in Alzheimer’s Disease

**DOI:** 10.3389/fimmu.2020.00754

**Published:** 2020-04-30

**Authors:** Courtney M. Kloske, Donna M. Wilcock

**Affiliations:** Department of Physiology, Sanders-Brown Center on Aging, University of Kentucky, Lexington, KY, United States

**Keywords:** microglia, cytokines, dementia, apolipoprotein E allele, neuroinflammation

## Abstract

Alzheimer’s disease (AD) is the most prevalent form of neurodegenerative disease, currently affecting over 5 million Americans with projections expected to rise as the population ages. The hallmark pathologies of AD are Aβ plaques composed of aggregated beta-amyloid (Aβ), and tau tangles composed of hyperphosphorylated, aggregated tau. These pathologies are typically accompanied by an increase in neuroinflammation as an attempt to ameliorate the pathology. This idea has pushed the field toward focusing on mechanisms and the influence neuroinflammation has on disease progression. The vast majority of AD cases are sporadic and therefore, researchers investigate genetic risk factors that could lead to AD. Apolipoprotein E (ApoE) is the largest genetic risk factor for developing AD. ApoE has 3 isoforms-ApoE2, ApoE3, and ApoE4. ApoE4 constitutes an increased risk of AD, with one copy increasing the risk about 4-fold and two copies increasing the risk about 15-fold compared to those with the ApoE3 allele. ApoE4 has been shown to play a role in Aβ deposition, tau tangle formation, neuroinflammation and many subsequent pathways. However, while we know that ApoE4 plays a role in these pathways and virtually all aspects of AD, the exact mechanism of how ApoE4 impacts AD progression is murky at best and therefore the role ApoE4 plays in these pathways needs to be elucidated. This review aims to discuss the current literature regarding the pathways and mechanisms of ApoE4 in AD progression with a focus on its role in neuroinflammation.

## Introduction

### Alzheimer’s Disease

Alzheimer’s disease (AD) is one of the most common neurodegenerative diseases and the 6^*th*^ leading cause of death in the United States. AD affects more than 5.7 million Americans and by 2050, it is projected to affect over 13 million. Not only is AD a growing health concern, it is also an extreme financial burden costing nearly 290 billion dollars, annually, not taking into account the thousands of unpaid caregivers (1, 2). Clinically, AD is characterized by progressive learning and memory deficits that ultimately impede a patient’s ability to perform daily activities. The hallmark plaque and tangle pathology associated with AD were originally described in 1907 by Alois Alzheimer. It is now known these plaques are composed of aggregated beta-amyloid (Aβ), and the tangles are composed of hyperphosphorylated, aggregated tau, present typically within the neurons. While these two hallmark pathologies together lead to the neurodegeneration seen in AD, (3–5) Aβ pathology deposition typically begins decades before tangles and tangles have been shown to be better indicators of cognitve decline (2, 6). Going back to Alois Alzheimer’s first description of the disease in 1907, he also noted activation of microglia and astrocytes in response to the pathology (5, 7–9), providing further areas in AD to study. In recent years, one of the major themes in AD research has been understanding neuroinflammation and the role it plays in AD progression using both animal models and human tissue.

While all cases of AD have Aβ plaques and tau tangles, the mechanism leading to pathology is believed to differ between cases. Exceptionally few cases (<1%) develop solely due to genetics, with mutations in genes involved in Aβ processing being the clear cause. Three genes where mutations are known to cause AD are the amyloid precursor protein (APP), presenilin 1 (PSEN1) and presenilin 2 (PSEN2) (10–12). Inheritance of any of these genetic mutations will lead to the accumulation of Aβ and ultimately AD. These cases are characterized as early onset AD (EOAD), affecting patients between 30 and 60 years of age (13, 14). The remaining 99% of AD cases are sporadic and are often associated with late onset AD (LOAD). Apolipoprotein E4 (ApoE4) stands out as the largest genetic risk factor for developing LOAD (15–17). Apolipoprotein (ApoE) has three isoforms with varying risk for developing AD. ApoE4 confers an increased risk of AD relative to ApoE3 with E4 homozygotes showing the greatest risk of AD with an odds ratio of 10–15-fold increase. ApoE4 is present in about 14% of the general population and 37% in the AD population (16–18). ApoE3 is the most common allele is typically used as the baseline comparison in AD studies. ApoE3 is present in about 78% of the general population and 59% in the AD population (16). The ApoE2 allele has been shown to be protective for AD compared to the ApoE3 allele, being present in about 5% of all AD cases and about 9% in the general population (19). It is important to note, studies have shown ApoE4 has an increased risk in AD and ApoE2 has been protective when comparing these changes to ApoE3, as it is used as the control allele (20).

### ApoE in the Central Nervous System

ApoE is the primary transporter of lipids and cholesterol in the brain and is mainly generated by astrocytes; however, microglia and neurons can generate ApoE in times of stress (21). ApoE works to reduce cholesterol levels as well as promoting lipoprotein clearance. ApoE binds to lipoproteins and provides clearance through the low-density lipoprotein receptor (LDLR). The ApoE isoforms impact how lipoproteins are cleared and the extent in which it is executed. ApoE3 has been shown to bind to LDLR allowing for lipid uptake and, again, it is characterized as the control phenotype for comparing function of the other two alleles. ApoE2 has a decreased affinity to LDLR and therefore leads to a type III hyperlipoproteinemia associated with ApoE2 patients. ApoE4 has an increased lipid binding ability but decreased proteolytic activity leading to an increase in lipoproteins and cholesterol (21–24). On astrocytes, ApoE interacts with plasma membrane ATP-binding cassette transporter A1 (ABCA1) and becomes loaded with lipids and cholesterol to provide the brain with needed nutrients (25–27). In addition to ApoE4 having an impaired interaction with receptors, ApoE4 has been shown to have a reduced interaction with ABCA1 and therefore is typically found in a hypolipidated state compared to ApoE3 (28–30).

ApoE4 has been suggested to decrease insulin signaling by impairing recycling of the insulin receptor which in turn could be leading to the decreased glucose metabolism seen in AD patients (31, 32). ApoE4 has also been shown to directly impact ApoER2 receptor recycling. This receptor works in conjunction with Reelin and is critical for synaptic plasticity in the aging brain. ApoE4 impairs recycling of ApoER2 and therefore impairs synaptic plasticity (33, 34). APP recycling has also been shown to be influenced by ApoE4 which can lead to an increase the amyloidogenic pathway leading to an increase in Aβ (35). In both post-mortem AD human brains and in mouse models of AD, ApoE4 has been shown to play a significant intracellular role in the movement and trafficking of receptors and intracellular vesicles (36).

The role of ApoE4 in the brain as a whole has been studied in metabolic approaches as well as through gross anatomy. These studies have implicated ApoE4 in increased regional cortical atrophy in the presence of AD compared to ApoE3. Specifically, there is a decrease in gray matter volume in both the medial temporal lobe and the anterior temporal lobes in AD cases (37–39). ApoE4 has also been implicated in overall brain energy and health as previously mentioned. Studies have shown ApoE status plays a role in cerebral glucose metabolism in an aging brain, regardless of the pathology present. At least one copy of ApoE4 significantly decreases glucose metabolism in comparison to non-ApoE4 carrying patients (40, 41).

ApoE has been shown to play integral roles in overall brain health and impact the development of Alzheimer’s disease. ApoE4 additionally plays many roles that are directly pertinent to the progression and development of AD specially through affecting inflammation. This will be discussed at length in this review.

## Inflammation in Alzheimer’s Disease

Neuroinflammation in AD has been shown to contribute significantly to the onset, progression, and pathogenesis of AD. The main sources of inflammatory mediators in the brain are microglia and astrocytes. Microglia and astrocytes can release cytokines which can play both pro-inflammatory and anti-inflammatory roles in the brain depending on the stimulus and microenvironment (7, 42, 43). This fluctuation between the pro- and anti-inflammatory profiles has be seen in patients with early AD. One study showed patients with early AD have a bias toward either a pro-inflammatory or anti-inflammatory phenotype and, as disease progresses, the phenotype is more homogeneous, with both sides of inflammation elevated relative to age-matched, non-disease controls (44). These findings demonstrate the complex, and dynamic nature of inflammation in AD. Neuroinflammation is also impacted through normal aging, however, this review will focus on the impact on AD. For more information on neuroinflammation and aging see the review from Rea et.al (45).

### Microglia in Alzheimer’s Disease

Microglia are the largest player in neuroinflammation in the CNS. Microglia have an important role in surveying the brain in order to detect and clear debris while maintaining an optimal microenvironment. Microglia can respond to virtually all foreign factors in the brain typically described as danger-associated molecular patterns (DAMPs) or pathogen-associated molecular patterns (PAMPs) (46–50). Of particular importance to AD, microglia respond to Aβ. Reports have suggested that in the presence of Aβ, microglia become activated and surround the plaque forming a barrier with the ultimate goal of preventing further spread while also attempting to clear the Aβ (51, 52). Upon activation around the Aβ plaque, the microglia can phagocytose Aβ (52, 53). However, if there is a buildup of Aβ in the microglia, this can subsequently lead to microglial cell death and an increase in inflammation and recruitment of more microglia, thus continuing this inflammatory cascade (54). Additionally, the activated microglia can respond with a pro-inflammatory response, releasing cytokines such as tumor necrosis factor-α (TNFα) and interleukin 1β (IL-1β) as well as other factors to potentially induce damage to surrounding tissue (50, 55–59).

Once a microglial receptor binds to a given ligand, microglia are able to become activated and work to ameliorate the situation. In regards to the clearance of Aβ, Aβ receptors have been shown to be present and have the ability to clear Aβ in early AD (60). In later stages of AD, however, this overall expression of both Aβ receptors and Aβ degradation enzymes are significantly downregulated. This downregulation of the Aβ receptors and Aβ degradation enzymes has been shown to be a direct response to the increase in inflammatory cytokines in AD (57, 61). One study looking at APP/PS1 mice found a twofold to fivefold decrease in Aβ receptors and a 2.5-fold increase in IL-1β and TNFα in older aged mice. This increase in pro-inflammatory cytokines can impair the surrounding neurons leading to neuronal degeneration (57). There are many microglial receptors that are able to bind Aβ and work to either phagocytize or create an inflammatory response to recruit other microglia in the presence of Aβ (62–65). Without control over their inflammatory response, the overall response of the microglia could become detrimental and cause problems such as neurodegeneration.

Microglial activation, in addition to phagocytosis and recruitment, can lead to induction of the inflammasome which can increase inflammatory cytokines as well as possibly increase Aβ deposition (59, 66). Inflammasomes help regulate the release of IL-1β and act as sensors of signals in the brain. IL-1β is incredibly potent, affecting the expression of adhesion molecules, immune cell infiltration and the overall increase of more cytokines. Due to its many functions, IL-1β requires several checkpoints before it is fully activated and, therefore, is made initially as an inactive molecule that the inflammasome cleaves and activates (67). The components needed for this activation include the inflammasome sensor molecule, adaptor molecule, and adapter protein apoptosis associated speck-like protein containing a CARD (ASC). Recent studies have shown, the ASC has the ability to help seeding of Aβ and by blocking the inflammasome it can decrease seeding of Aβ in the brain of mouse models (59, 67–71).

Upon debris clearance, microglia rapidly work to counteract their previously pro-inflammatory response (72–75). This can be done through the induction of anti-inflammatory responses which work to downregulate the potent, pro-inflammatory response. This allows for the microglia to revert to a resting state (50). In the case of AD, the microglia can become chronically activated and be in a perpetually activated state, leading to detrimental effects such as an increase in Aβ production and neurodegeneration (74, 76). In AD, both pro- and anti-inflammatory microglia states can be found in the same region, and this co-existence is likely detrimental; however, deciding which state the microglia should be in at a given instance remains unclear (77).

Recently, a microglial receptor, triggering receptor expressed on myeloid cells 2 (TREM2) has brought the importance of microglia in AD to the forefront of inflammatory research since mutations in TREM2 increase the risk for AD. As its name states, TREM2 is expressed on myeloid-derived cells such as microglia, macrophages, and osteoclasts. Rare mutations in TREM2 confer an increased risk of AD with an odds ratio of 4.5 (78–80). Individuals homozygous for mutations in TREM2 develop a rare disorder characterized by bone fractures and presenile dementia called Nasu-Hakola disease (81, 82). TREM2 responds to a wide range of stimuli including apoptotic cells, Aβ, and lipoproteins. Without AD pathology, TREM2 is expressed to clear damaged or apoptotic neurons and clear cellular debris through phagocytosis while downregulating the pro-inflammatory response to these stimuli (83–85). In AD, TREM2 has been shown to be highly expressed on microglia surrounding neuritic plaques and is important in clearance of Aβ (86, 87). In mouse models lacking TREM2, the microglia are unable to migrate toward an Aβ plaque and do not cluster when compared to a model with functional TREM2. While the overall impact of TREM2 is still up for debate, evidence suggests that the timing of TREM2 expression is key. Studies show that in early AD pathology, TREM2 is needed for clearance of early Aβ plaques and slowing of cognitive decline, while expression of TREM2 later in disease progression could lead to detrimental long-term consequences (88–92).

### Astrocytes in Neuroinflammation

**FIGURE 1:**
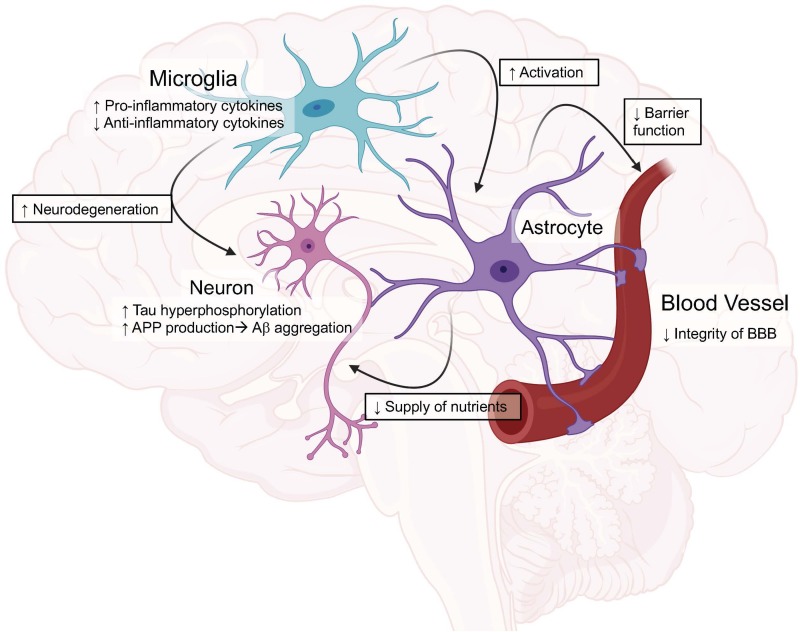
Graphical view of the impact of ApoE4 in the brain compared to ApoE3. These roles will be discussed in depth throughout the review. Created with BioRender.

Astrocytes also play a role in the inflammation seen in AD (93). In the brain, astrocytes interact with both neurons and the cerebrovascular in order to maintain nutrients and chemical gradients in the brain. They additionally work to maintain calcium levels and potassium homeostasis, as well as provide overall neuronal support. In AD, normal functions of astrocytes become affected and contribute to astrocytic dysfunction and inflammation. This dysfunction includes astrocytes losing their ability to maintain calcium levels and potassium homeostasis (94–96). Additionally, activated astrocytes typically lose their ability to deliver nutrients to neurons leading to impairments in neuronal function (97, 98). Upon activation, studies have shown astrocytes release cytokines that can lead to increased neuronal toxicity as well as a decreased outgrowth of neuronal processes and an overall decreased activity rate (99). Recent studies suggest an interaction between microglia and astrocytes finding that once microglia become activated, they can lead to activation of astrocytes, resulting in a feed-forward loop which is detrimental to the surrounding environment. The mechanism showed that when activated, microglia release IL-1α, TNFα and C1q and astrocytes become activated. Additionally, in the presence of damaged blood vessels, the astrocytes promote tissue repair and neuronal survival rather than microglia (99). This provides a strong link showing that both microglia and astrocytes work in conjunction to increase the neuroinflammation seen in AD (Figure 1).

## ApoE, Inflammation, and Alzheimer’s Disease

### Early Investigations of ApoE Isoforms’ Role on Inflammation

While microglia and astrocytes are the main modulators of inflammation in the brain, they are also the major sources of ApoE in the brain. Early studies suggested that in glial cultures, the presence of ApoE helped suppress glial activation in response to lipopolysaccharide (LPS), showing ApoE provides a protective anti-inflammatory response (100, 101). Studies showed that this anti-inflammatory response could be through the inhibition of the c-Jun N-terminal kinase (JNK) cascade (102). Another study showed that APP was capable of activating microglia and producing neurotoxic molecules in the presence of ApoE4, however ApoE3 was able to prevent this activation (49).

Moving into animal studies, researchers then began to investigate how ApoE as a whole, as well as the isoforms, impacted neuroinflammation (49, 103, 104). In one study, APP mice with and without ApoE were chronically administered LPS and it was found that in the presence of ApoE, the mice had increased gliosis and Aβ deposition suggesting a role for ApoE to increase inflammation in AD models (104). When looking at the ApoE isoforms specific impact on the inflammatory response, a study from Lynch et.al. used humanized ApoE3 and ApoE4 mice and chronically administered LPS. They found a significant increase in TNFα and IL6 present in the brains of ApoE4 mice. The authors then examined the impact of a small ApoE-mimetic peptide on inflammation and saw a significant reduction in inflammation. These results together show ApoE isoforms impact inflammation in the brain and that exogenous ApoE has the potential to reverse these effects (105). Another study using humanized ApoE3 and ApoE4 models showed ApoE isoforms play a regulatory role in nitric oxide (NO) production from microglia. They show that ApoE4 microglia release significantly more NO compared to ApoE3 microglia, which could cause the detrimental effects seen in the brains of ApoE4 patients (106). The results from this study were later confirmed and further research was done showing ApoE4 increased NO production in mice as well as in humans. Additionally, the authors showed resting microglia from the ApoE4 targeted replacement mice had an increased proinflammatory profile which led to an altered microglial phenotype compared to ApoE3 (Figure 1) (107).

### ApoE and Aβ

Evidence indicates that the three common ApoE isoforms impact the clearance and aggregation of Aβ (16, 35, 108–112). Studies have shown that ApoE is essential for deposition of Aβ in animal models (29, 111, 113–115). ApoE4 has been shown to have an increased binding ability to Aβ compared to ApoE3 which contributes to the increased aggregation of Aβ and decreased clearance. In addition to increased binding, ApoE4 has been shown to have increased rate of Aβ oligomerization and increased Aβ plaque generation (116, 117). This increase has been confirmed in human autopsy tissue as ApoE4 patients show an increase in both vascular and parenchymal Aβ plaques (37, 118–120). While the exact mechanism of how ApoE4 carriers exhibit increases in Aβ plaques is not fully understood, it may be due to the increased ability of ApoE4 to bind Aβ and inability to fully remove Aβ from the brain.

**FIGURE 2:**
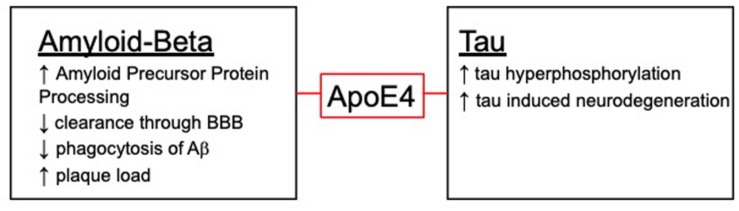
The role ApoE4 plays with the hallmark AD pathologies.

There are several mechanisms in which Aβ could be cleared and where ApoE4 could impact clearance (Figure 2). Typically, Aβ is removed through either proteomic degradation, lysosomal clearance or through the blood brain barrier (BBB) (121). ApoE4 has been shown to have an impact on these pathways and could contribute to the decrease in Aβ removal seen with the ApoE4 isoform. In addition to clearance of Aβ, ApoE has also been shown to play a role in phagocytosis of apoptotic cells which is necessary in AD due to neurodegeneration. Surprisingly, one study showed ApoE4 has an increased phagocytosis of apoptotic neuronal cells while having a decreased clearance of Aβ *in vitro*. This increase in phagocytosis could be contributing to the switch in phenotype from resting microglia to an altered phenotype, leading to the detrimental phenotype associated with ApoE4 (122).

APP processing and recycling has also been shown to be influenced by ApoE4, leading to an increase in Aβ (35). A recent study showed ApoE isoforms differently regulated APP processing through altered MAPK signaling. They showed that ApoE4 can function as a signaling molecule to enhanced activity of this cascade leading to an increase of APP. This study gives an ApoE dependent mechanism in which ApoE4 could directly impact disease progression through alteration in a signaling cascade (123).

In addition to ApoE4 playing a role in Aβ generation, ApoE4 and Aβ can also have a significant role in the ApoE-inflammatory cascade. As previously mentioned, ApoE4 has a role in increasing the proinflammatory response of glia in response to inflammatory stimuli. Researchers then moved into transgenic mice expressing human ApoE in various AD models to determine (1) the role of ApoE and (2) the role of ApoE isoforms in disease progression. In a study investigating the impact of ApoE using APP/PS1 mouse models, researchers found ApoE had a direct impact on the deposition of Aβ through its influence on microglial activation. These results show ApoE is required to stimulate the innate immune response to Aβ (124). A study using human ApoE mice with familial AD mutations (EFAD model), found ApoE4 significantly impacted Aβ plaque morphology and showed increased glial activity. The increased glial activity was measured by IL1β levels and showed a negative impact on microglial morphology (114).

### ApoE and Tau

ApoE4 has been shown to have varying inflammatory effects not only on Aβ but, it has been shown to have differential effects on tau induced neuroinflammation (Figure 2). ApoE4 has been shown to exacerbate neurodegeneration through a tau-mediated mechanism using P301S-ApoE TR models. The authors showed ApoE4 microglia generate significantly more TNFα and led to impaired neuronal viability compared to other ApoE isoforms (125). Another study described how microglia could be playing a vital role in neurodegeneration in a similar tau model through an ApoE dependent method. This study suggests microglia could be a key target in therapeutics when it comes to tauopathies (126). This idea of microglia and ApoE being the driving factor in the tau deposition is paralleled in another study using a model of microglial ablation. This study showed microglia were the key player in promoting plaque formation and neurodegeneration in the animal models (127). These studies show the importance of investigating both the detrimental and beneficial roles of microglia, especially with ApoE isoforms (Figure 1).

### ApoE and Inflammatory Signaling Cascades

ApoE has been shown to interact with TREM2 leading to activation of TREM2’s signaling cascade (91, 128–130). Recent studies have suggested that, in the presence of neurodegeneration, ApoE and TREM2 are both required for microglia to become activated and assume a Disease Associated Microglia (DAM) phenotype. This phenotype allows for microglia clustering around the Aβ plaque and clearance of apoptotic neurons. Microglia with a DAM phenotype are highly localized around neuritic plaques and activation requires a step wise transition (131–134). First is an increase in *Apoe* and downregulation of homeostatic genes such as Purinergic Receptor P2Y12 (*P2ry12)*, followed by an upregulation of *Trem2*. This DAM phenotype has helped bring together ApoE and TREM2 through a disease specific pathway as well as how mulitple risk factors for AD play a role in this cascade (131, 134). Further studies are needed to examine the role of ApoE isoforms on this cascade as well as looking into the proteomics of these cascades.

### ApoE and Autophagy

Autophagy is a mechanism to eliminate aggregated proteins and internal structures that become dysfunctional. Studies have shown this pathway specifically becomes dysfunctional early in disease states and can lead to neurodegeneration. With the role of ApoE4 in neurodegeneration, researchers have suggested that ApoE4 likely has an impact on clearance of neuronal degenerative products after injury since ApoE is upregulated post insult, thus potentially linking ApoE4 with autophagy dysfunction (135–138). Recent studies suggest a potential interaction with ApoE4 and an autophagy pathway that may account for the decreased autophagy in ApoE4 carriers (139). This study showed a direct interaction with ApoE4 and coordinated lysosomal expression and regulation (CLEAR), suggesting ApoE4 has a direct impact on autophagy transcription leading to impaired autophagy (140). This allows for further studies in ApoE4 as a transcription factor as well as other potential mechanisms in which ApoE4 impacts autophagy.

### ApoE Fragmentation

The ApoE4 protein has been shown to have a decreased expression compared to the other isoforms but the exact mechanism of this is unknown. One possibility of this downregulation is through the rate of degradation of ApoE. Studies have suggested that ApoE can be fragmented by high-temperature requirement serine peptidase A1 (HtrA1). Of the ApoE isoforms, ApoE4 is the least stable protein confirmation and therefore most susceptible to this fragmentation. These fragments could bind to Aβ or cell surface receptors to prevent clearance, possibly contributing to the progression of the disease through neurotoxicity and neuroinflammation (141–144). ApoE3 also can become fragmented and studies suggest the primary fragment produced is neuroprotective (105, 145). More studies are needed to examine the exact mechanism in which ApoE4 fragments can contribute to inflammation and disease progression and potential therapeutics with ApoE3 fragments.

### ApoE and Blood Brain Barrier Dysfunction

The importance of ApoE on the BBB has been a growing topic in the field of AD as well as other brain injury research due to the fact that brain trauma leads to a disruption in the BBB (146). Studies have shown patients with ApoE4 have worsened outcomes to brain injuries including traumatic brain injury (TBI) and stroke compared to ApoE3. Additionally, TBI earlier in life has been shown to contribute to an increased risk of AD later in life. TBI patients with ApoE4 typically experience increased coma length, mortality and decreased prognosis (147–150). These suggest ApoE isoforms play a role in BBB regulation and ApoE4 has a clear impact on the overall strength and integrity of the BBB (Figure 1).

The mechanism as to how ApoE isoforms could be involved in BBB dysfunction is necessary to understand why ApoE4 causes detrimental outcomes when the brain becomes compromised. One crucial component of the BBB are the tight junctions, as they provide a barrier that allows selective molecules to flow between blood and brain, maintaining homeostasis and keeping out unwanted molecules. Nishitsuji et al. showed that these tight junctions are important in BBB integrity and are significantly impaired in the presence of ApoE4. The authors suggest that this could be due to changes in matrix-metalloproteinase-9 (MMP9) or other molecules that would degrade or impact the BBB integrity (151). MMP9 has been shown to have multiple targets present in the BBB, but one specifically is tight junctions. MMP9 can work to decrease the bond in the tight junction leading to a leaky BBB (152). A study from Bell et.al built upon the idea that ApoE4 triggers BBB breakdown and found that *cyclophilin A (CypA*) is the likely culprit in this breakdown. They showed that an increase in *CypA* led to activation of NF-kB, followed by a significant increase in MMP9, specifically in the pericytes found surrounding and supporting the vasculature in the brain (153). Recently, Main et.al showed that ApoE4 had an impact on the BBB integrity and the activation of the MMP9 pathway in a comprehensive study using a TBI model. This study once again confirmed that ApoE4 with TBI worsens disease outcomes. They also found that after injury in both ApoE3 and ApoE4, the BBB must become stabilized which leads to an increase in ApoE. After the ApoE increase, there is pericyte loss, decreased tight junction expression and an increase in MMP9 expression. This appears in both ApoE3 and ApoE4 models, however, ApoE4 is much slower at the resolution of this progression. ApoE4 has clear detrimental effects on BBB integrity by activating the MMP9 system and activating other inflammatory cascades (154). In the presence of ApoE4, the pericytes significantly lose their supportive capabilities, leading to an increase in leaky vessels (155). A culmination of these studies implicates ApoE4 in decreasing BBB integrity through weakening tight junctions, increases in MMP9 and a loss of support needed for the BBB.

The evidence shows an increase in MMP9 with ApoE4 leads to the loss of BBB integrity and increase in inflammation. ApoE4 has also been associated to an increase in cerebral amyloid angiopathy (CAA) which can trigger BBB dysfunction due to the deposition of Aβ in the vessel walls and impaired clearance through the BBB compared to ApoE3 (118). Anti-Aβ immunotherapies for animal models and in clinical trials have been shown to increase MMP9 expression leading to an increase in microhemorrhages, showing a decrease in BBB integrity (156). While the majority of the anti-Aβ trials failed due to adverse effects, these adverse events were more robust in ApoE4 carriers. This connection between MMP9 activation due to the anti-Aβ therapies and the effect of ApoE4 on MMP9 expression can explain the ApoE4 involvement in BBB dysregulation that in turn, leads to neurodegeneration. More information on ApoE4 in clinical trials will be provided later in the review.

In addition to the disruption of the BBB integrity upon brain injury, there has been shown to be an interesting connection between TBI and development of AD later in life. In ApoE TR models of ApoE3 and ApoE4, the mice were given repeated TBI over 1 month. This showed a significant increase in microgliosis, via IBA1 immunoreactivity, in ApoE3 mice but not ApoE4 mice. ApoE4 mice showed significant increases in phospholipids and LDLR but not in inflammation suggesting that ApoE4 plays a role in many detrimental pathways but also shows an impaired inflammatory phenotype (157). This finding suggests that something more than just a decrease in BBB integrity is occurring in the presence of ApoE4.

### ApoE and the Glymphatic System

As previously discussed, ApoE4 constitutes a significant impairment in the clearance of Aβ. This leads to the buildup of Aβ in the vessels of ApoE4 AD patients, contributing to an inflammatory response. This impairment in clearance has been hypothesized to be directly related to ApoE4 forming globular structures in conjunction with Aβ, potentially impairing the availability of receptors necessary for the clearance of Aβ into the blood vessels. Due to the involvement of ApoE in clearance of Aβ from the brain to the vasculature, it is logical to hypothesize that ApoE4 could also be involved in the impairments seen in the glymphatic system due to a similar mechanism. Studies thus far have examined glymphatic system impairments with Aβ removal without examining ApoE isoforms. This study also examined human autopsy tissue and found impairments and buildup of Aβ in the glymphatic system but again, ApoE isoforms were not considered (158, 159). More studies are needed to specifically examine human tissue and consider ApoE isoforms to determine the potential role of ApoE in the glymphatic system.

### ApoE and Other Neurodegenerative Diseases

Studies have implicated ApoE4 in other forms of dementia other than AD, including Lewy Body Dementia (LBD). LBD is one of the most prevalent forms of dementia and is found in about 40% of Parkinson’s Disease patients. Two independent studies published in 2020 investigated the effect of ApoE4 on α-synuclein (α-syn), the defining pathology seen in LBD, and showed an increase in pathology was associated with ApoE4. Both studies used transgenic mice expressing human ApoE isoforms with differing routes of α-syn production (transgenic model and AAV- α-syn). The results from the animal studies both showed ApoE4 lead to an increase in α-syn pathology, a decrease in cognitive decline and an increase in gliosis around the pathology (160, 161). In addition to animals, human patients were also investigated, and it was shown that ApoE4 patients with Parkinson’s disease had an increased rate of cognitive decline (161). Human neuropathology data showed that ApoE4 patients had an increase in α-syn pathology as well (160). Together these studies show a clear impact of ApoE4 in other neurological diseases as well as the impact of ApoE4 on neuroinflammation is not limited to AD pathology.

### Impact of ApoE on Clinical Trials

Due to the growing impact of AD, the need for therapeutics to either prevent or delay the onset of AD is imperative. In 2012, the National Alzheimer’s Project Act was established to help prevent future cases of AD and related dementias. The group came up with five major goals that includes optimizing care quality for patients, improving support for those with AD and their families, enhancing public awareness, tracking progress, and preventing and effectively treating AD by 2025 (162). Having a prevention or treatment option by 2025 is a lofty goal but the field is working hard to achieve this goal. While we have had many drug trials in the past 20 years, the failure rate is high which is pushing the field in a direction of a more personalized medicine approach to AD. Using this idea in clinical trials and therapeutic treatments, the likelihood of a single drug helping everyone with the disease is not likely. As this review has showed, ApoE4 plays a large and varied role in AD, and thus is necessary to take into consideration upon selection for clinical trials.

Targeting Aβ through anti-Aβ immunotherapy has been a constant focus of trials and is still being actively perused. Bapineuzumab was one of the initial anti-Aβ immunotherapies designed to increase clearance of Aβ. In a phase II clinical trial, bapineuzumab treated ApoE4 non-carriers had significant benefits on cognition and function at the endpoints of the study while this was not seen in ApoE4 patients. This trial also showed that ApoE4 patients had an increase in amyloid related imaging abnormalities (ARIA) which occurred 3–7x more in ApoE4 patients depending on copy number. These findings led to the phase III trial where ApoE4 patients received a lower dose to offset ARIA (163–165). This study ultimately failed to meet the endpoints and Aducanumab moved in as a newer anti-Aβ immunotherapy for trial. Aducanumab can be tolerated at a higher concentration and was dosed every 4 weeks. This trial was focused on patients with AD before cognitive symptoms had occurred. While the drug showed an effect on cognition, ARIA was the main adverse effect in ApoE4 patients. Moving into the phase III trial for Aducanumab, patients were segregated out into ApoE status to dose according (166). These two studies have clearly shown that while the goal is to find a treatment for AD, it is important to consider ApoE status into the trial design and study outcomes.

Currently, very few clinical trials take ApoE status into account while grouping or dosing for trials. One current ongoing trial, “A Study of CAD106 and CNP520 Versus Placebo in Participants at Risk for the Onset of Clinical Symptoms of Alzheimer’s Disease” (NCT02565511), is investigating the effects of CAD106 and CNP520, which both target amyloid, on the impact of cognition, clinical status and pathology. They will investigate ApoE4/4 patients due to their high risk of progression to MCI and AD and is expected to reach completion in 2024. Another upcoming trial is directly targeting ApoE4 patients by using an adeno-associated virus in order to convert ApoE4 patients to ApoE2 in hopes to delay AD onset (NCT03634007). Studies incorporating ApoE status into their inclusion and dosing criteria will be imperative in the growing field of AD trials and research.

One of the most multi-faceted clinical trials going on right now is the Finnish Geriatric Intervention Study to Prevent Cognitive Impairment and Disability (FINGER), which now has branches in the United States as well as around the world. The original FINGER study showed that after lifestyle modifications, ApoE4 carriers showed no significant cognitive improvements to those without ApoE4 and that E4 carriers with lifestyle modifications had greater cognitive and physical improvements than those without the modifications (167, 168). This study emphasized the importance of preventative strategies for ApoE4 patients, specifically through lifestyle modifications such as physical activity.

## Conclusion

As shown through this review, ApoE4 plays a role in virtually all aspects of AD ranging from clearance of hallmark pathologies, to disruption of intracellular pathways, to impacts on whole brain metabolism. While these pathways can seem unrelated, they can be connected through the overarching theme of neuroinflammation. In moving forward, the impact of ApoE4 needs to be considered in studies, both human and animals. In conclusion, ApoE4 plays a negative role in AD through both gain of misfunction and loss of function mechanisms and further studies are needed to elucidate these pathways and push the field toward new therapeutics.

## Author Contributions

CK wrote and developed much of the manuscript content. DW wrote the portions, edited, and reviewed for content.

## Conflict of Interest

The authors declare that the research was conducted in the absence of any commercial or financial relationships that could be construed as a potential conflict of interest.
